# Exploring Digital Vā: A Scoping Review of Conceptualisations of Vā in Digital Spaces

**DOI:** 10.1002/snz2.70080

**Published:** 2026-09-10

**Authors:** Jean M. Uasike Allen, Joanna Ting Wai Chu, Latesha Latu, Analosa Veukiso‐Ulugia, Carine Umutoniwase, David Taufui Mikato Fa’avae, Jacoba Matapo, Lawrence May, Kate Jack

**Affiliations:** ^1^ School of Education and Social Practice Faculty of Arts and Education Waipapa Taumata Rau, University of Auckland Auckland New Zealand; ^2^ School of Population Health Faculty of Medical and Health Sciences Waipapa Taumata Rau, University of Auckland Auckland New Zealand; ^3^ Office of Pacific Advancement Auckland University of Technology (Te Wānanga Aronui o Tāmaki Makaurau) Auckland New Zealand; ^4^ Faculty of Arts and Education Waipapa Taumata Rau, University of Auckland Auckland New Zealand

**Keywords:** digital vā, online communities, online gaming, Pacific values, Pacific youth, well‐being

## Abstract

The well‐being of young people globally is a key concern for many. In Aotearoa New Zealand, Pacific people are the youngest ethnic population, and Pacific youth experience complex well‐being challenges related to socio‐economic inequality and diverse identities. However, recent research indicates that Pacific youth are navigating new forms of social and cultural connection, including in digital spaces. This article presents the findings of a scoping review focused on digital vā, a pan‐Pacific relational ontology, and its relationship to well‐being, applied to an online gaming context. Conducted according to the Preferred Reporting Items for Systematic Reviews and Meta‐analyses (PRISMA) guidelines, 670 studies were identified, of which 17 met the inclusion criteria. Findings indicate a scarcity of literature focused on Pacific youth, digital vā and online gaming, underscoring the need to explore how Pacific youth engage emerging digital spaces in relation to their well‐being from their own perspectives in a future‐focused manner.

## 1 Introduction

Pacific people in Aotearoa New Zealand (NZ) comprise the country's youngest ethnic group, with 58.7% of the total Pacific population in 2023 being 29 years old or younger (StatsNZ 2024). As a steadily increasing population vital to the economic, social and cultural future of Aotearoa NZ, calls are growing for health research to focus on Pacific youth well‐being (Health Quality & Safety Commission [HQSC] 2021; Veukiso‐Ulugia et al. 2024).

While a recent national study of Pacific youth found that, generally, Pacific youth are happy, healthy and report positive social relationships, it also identified serious inequities that affect Pacific youth at higher rates than most other ethnic groups (Veukiso‐Ulugia et al. 2024). In 2019, 25% of Pacific youth reported significant depressive symptoms (Fleming et al. 2020), and over 25% reported being unable to access appropriate healthcare when needed (Veukiso‐Ulugia et al. 2024).

Pacific youth are particularly vulnerable to health risks due to disproportionate exposure to poor socio‐economic determinants of well‐being. Almost two thirds of Pacific youth lived in high‐deprivation neighbourhoods in 2019, and almost half experienced serious housing insecurity (HQSC 2021; Veukiso‐Ulugia et al. 2024). Pacific youth also report unique struggles navigating complexities associated with multi‐ethnic identities and the hegemonic Pākehā cultural context of NZ. In 2019, 40.5% reported experiences of racism and others described feelings of cultural disconnectedness (Fleming et al. 2020; Veukiso‐Ulugia et al. 2024; Wilson and Nicolson 2020).

Although research suggests that COVID‐19 exacerbated existing well‐being inequities for Pacific youth due to social isolation (Laulaupea’alu 2021), it also reveals how Pacific people, and especially Pacific youth, have been using digital technology in innovative ways for connection and communication since well before the onset of the pandemic (Enari and Matapo 2020; Thomsen et al. 2021). As a result, Pacific healthcare leaders have suggested that exploring how digital spaces impact Pacific youth well‐being, from a youth‐led perspective, will be necessary to improving well‐being outcomes (HQSC 2021). Examining these factors will be critical for developing protective initiatives and structures and uplifting the resilience and agency of Pacific youth.

As Teariki and Leau (2023) have suggested, the worldviews that underpin established Pacific health and well‐being models may not always reflect those of youth. Pulotu‐Endemann (2001) remains the most widely accepted Pacific model of health. The Fonofale Model, based on the structure of a Samoan house, articulates well‐being as influenced by time and context. Therefore, understandings of health and well‐being change across time, space and place, especially for Pacific youth who may experience the world differently to their elders, particularly within digital spaces (Koya Vaka’uta 2017; Lopesi 2018). Online, various factors are likely to impact the well‐being of different Pacific communities, such as a spatial‐generational divide in digital access and literacy (Kemp 2021). For instance, despite concern that participation in online gaming has negatively affected the educational outcomes and mental health of young people (e.g., Brannam 2021; Stewart et al. 2019), research with ethnically diverse youth suggests instead that gaming can facilitate social support and community‐building (Kelly et al. 2021; Militello et al. 2018).

As youth innovate new ways to use digital spaces (HQSC 2021; Koya Vaka’uta 2017; Lopesi 2018), established models of health may not always account for these experiences. For example, the Fonofale Model is popular among Pacific academics and communities but was created by adults more than 20 years ago. While there are limited critiques of this model, Galuvao (2016), questions its versatility across all Pacific groups, raising questions about its relevance to youth and the potential exclusion of diverse voices from communal understandings of well‐being (Veukiso‐Ulugia et al. 2025). Thus, despite researchers’ intentions, a lack of Pacific youth voice in health research can limit understanding of the relationship between well‐being and digital space.

Digital vā is an increasingly popular concept that explores relationality online by considering vā, a cornerstone of Pacific cultural practice regarding the building and maintaining relationships, in digital terms (Ka’ili 2017; Thaman 2008; Enari and Matapo 2020). Vā refers to the spatialised interaction and relationships between people and their environment as enacted through the expectations and values of each Pacific culture. As a relational Indigenous Pacific ideal, it opposes Westernised humanist onto‐epistemic frameworks (Enari and Matapo 2020; Matapo 2022). Well‐being is connected to vā as a relational ontology—fully and appropriately sustaining relationships is critical to maintaining health and well‐being.

Though we initially focused on reviewing the concept of digital vā relative to online gaming, limited discussion of these specific ideas demanded that we broaden our focus. As a result, this scoping review highlights research on digital vā and well‐being to develop understanding of the existing nature of the term, responding to Lopesi's (2018) suggestion that ‘to understand Moana peoples in the twenty‐first century, we have to do a better job of understanding the significance and power of the online environment and understanding ourselves moving alongside technological developments’ (p. 103). We find that existing literature contributes to conceptualisations of Pacific youth well‐being while also asserting the legitimacy of Indigenous epistemologies that focus on youth engagement of under‐researched online spaces.

## 2 Methods

A scoping review was conducted according to the Preferred Reporting Items for Systematic Reviews and Meta‐Analyses extension for Scoping Reviews (PRISMA‐ScR) (Triccio et al. 2018). The review sought to identify studies conducted about digital vā and how it has been imagined and used, including in relation to well‐being. The scoping objectives included:

i. To identify and describe the current literature focused on digital vā.
ii. To gain insight into existing models, frameworks, and conceptual frameworks used alongside digital vā.
iii. To understand how well‐being is conceptualised relative to digital vā.

### 2.1 Search Strategy and Eligibility Criteria

A university librarian reviewed the search strategy, and it followed Tricco et al.'s (2018) PRISMA‐ScR guidelines. Electronic searches were conducted from 1st March until 4th April 2023 across four databases (*n* = 506: Science Direct, Web of Science, EBSCOhost and Scopus). The databases were chosen to provide a broad, interdisciplinary scope. The review was supplemented with a targeted hand search of key journals in Indigenous and Pacific research (*n* = 156: Journal of Indigenous Well‐being, Journal of Polynesian Society, MAI Journal and Journal of Pacific Studies). We also included grey literature searches through ProQuest Dissertations and Theses and the University of Auckland Library (*n* = 12), the first 100 results from Google Scholar and conducted reference mining of eligible studies to capture additional relevant work outside traditional databases.

The search term combinations were informed by the primary key term ‘digital vā’ and included additional terms such as ‘vā relationality’, ‘online vā’ and ‘vā relations’, as seen in Table 1. Articles were included or excluded based on the following criteria: They needed to be relevant to the Indigenous Pacific concept of digital vā, published in English between 2013 and 2023, and the full article was accessible for review. The search strategy concentrated on empirical studies and grey literature.

**TABLE 1:** Study search terms.

Key term	Search terms
Digital vā	Vā relationality or online vā or vā relations or digital vā

It should be noted that using the term ‘digital’ in the search attracted many studies related to computer technology use that did not align with the concept of online relationality among Pacific communities. This increased the number of results yielded from the electronic searches to 674 studies, of which 96% were unrelated to digital vā. Databases were also not able to read the macron in vā, leading to results that recognised vā as ‘veteran affairs’, ‘virtual assistance’ and ‘vertebral artery’, among others. Following the initial database search, title and abstract screening were conducted to refine the search by excluding records outside the review scope. This step was important for removing identified records where the term vā had these meanings or applications unrelated to a Pacific context.

### 2.2 Study Identification, Selection and Screening

**FIGURE 1:**
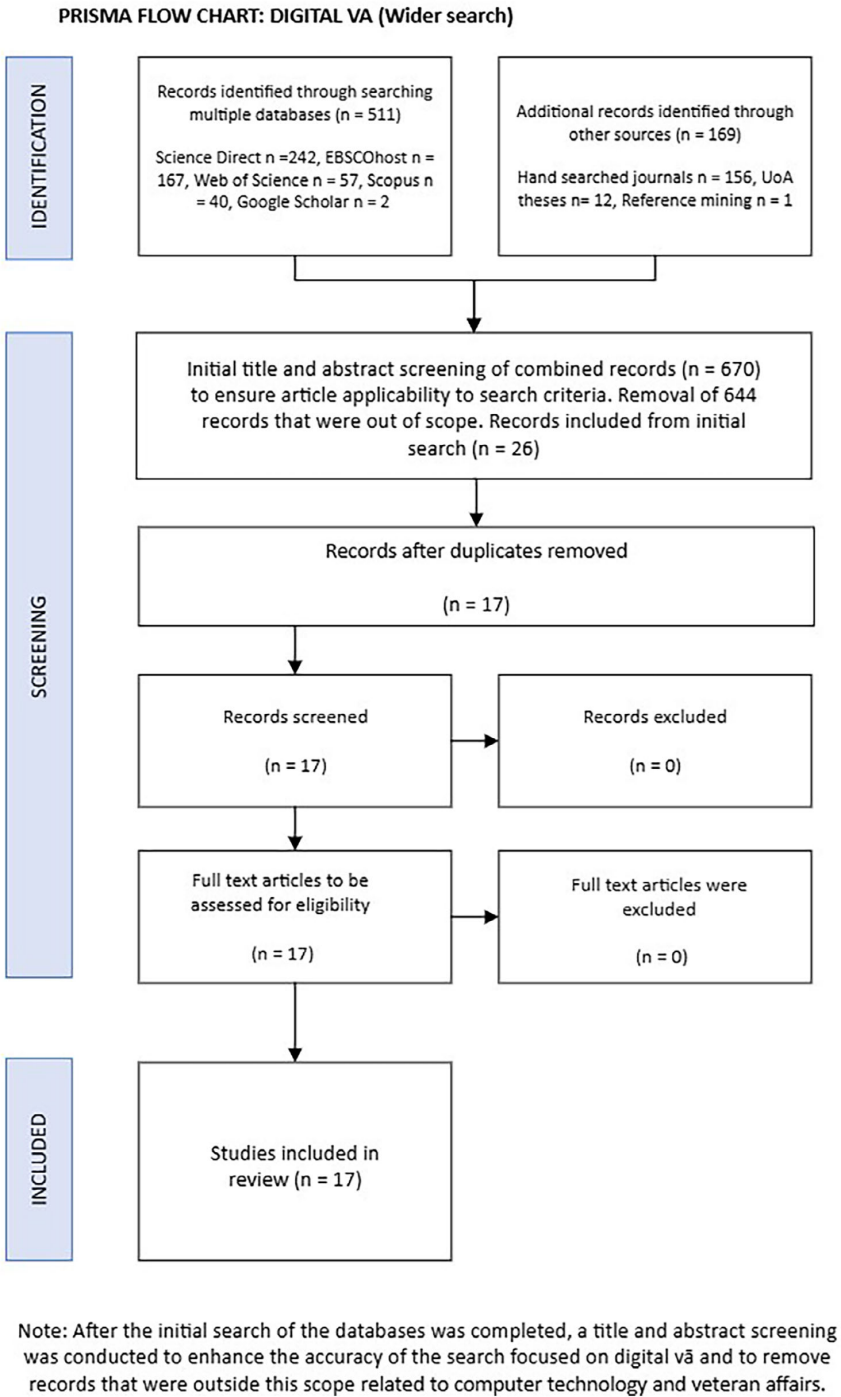
PRISMA flow chart for digital vā (wider search).

Study identification followed the prescribed eligibility criteria and is outlined in Figure 1. Identified studies (*n* = 670) went through a title screening process to ensure article applicability to the criteria. Title and abstract screening were conducted separately to narrow included articles to those focused on digital vā. As articulated previously, key search terms resulted in several articles inconsistent with the topic, so additional screening for accuracy was completed after the initial search and prior to removal of duplicates.

Title and abstract screening of initial results resulted in 26 studies identified across four databases (EBSCOhost *n* = 7, Web of Science *n* = 1, Scopus *n* = 6, Google Scholar *n* = 5), two journals (Journal of Indigenous Well‐being *n* = 5, MAI Journal *n* = 1) and one from reference mining. Articles from the Journal of Pacific Studies were already captured by other databases.

Abstract screening conducted independently by two reviewers removed nine duplicates, resulting in 17 studies being subject to full‐text screening. All 17 studies met the inclusion criteria and were subject to full‐text analysis. Any disagreement was discussed among all reviewers until a consensus was reached.

### 2.3 Data Extraction and Synthesis

Using Microsoft Excel, a data extraction table was developed based on the characteristics and variables of each study as related to the research objectives, as illustrated in Table 2.

**TABLE 2:** Characteristics of included studies.

Title	Author/s	Year	Location	Purpose	Study type	Study design	Sample	Outcome	Instrument	Analysis and key findings
Searching for the Digital Fāgogo: A Study of Indigenous Samoan Storytelling in Contemporary Aotearoa Digital Media	A. J. Tielu	2016	New Zealand	Exploring what fāgogo looks like in contemporary NZ media	Mixed methods	Talanoa, fāgogo, case study	Collected data about fāgogo from interviewees and online	How often fāgogo was effectively utilised in mainstream media, and what its use resulted in		There are two primary types of fāgogo: one that references Indigenous oral traditions and history and another that reflects contemporary life. Online fāgogo can be removed from cultural contexts, and contemporary fāgogo online can fail to perform as a fāgogo if it does not use digital spaces in a culturally responsive way
The Digital Vā ‐ Negotiating Socio‐spatial Relations in Cyberspace, Place and Time	C. F. Koya Vaka’uta	2017	Fiji	Examining the impact of social media on Pacific rules for social engagement, and whether Pacific people are digitising vā	Qualitative	Case study of intergenerational family guided by values theory	Convenience sampling (family)	How a family translates vā online, and how this differs from real‐time vā	Observations and note‐ taking	Analysed with guidance from values theory. Finds that digital vā and real‐time vā are distinctly different. Attempts to develop vā in the modern day are being transformed in various ways
The Digital Vā: Pasifika Education Innovation during the COVID‐19 Pandemic	D. Enari, J. Matapo	2020	Australia, New Zealand	Exploring the innovation and persistence of Pacific people during COVID‐19	Qualitative	Situation report				Digital tools are not new to Pacific people who have been using them to support the creation of international vā, and learners and families have similarly adapted in educational contexts throughout COVID‐19
COVID‐19 Muddles Talanoa and Vā: Perceived Connections and Uncertainties	S. Laulaupea’alu	2021	New Zealand	Considering the impact of ICT learning programmes on vā in higher education	Qualitative	Talanoa				Cybersecurity concerns and un/ethical use of online learning software can create challenges for maintaining vā in higher education spaces
Negotiating the Digital Vā: Empowering Pacific Scholars and Community Building on Twitter	P. Thomsen, L. Lopesi, G. Pōmaika’i Gushiken, L. Damm K. Lujan Lee, E. Pickering‐ Martin, F. Iosefo, S. Naepi, L. Tuiburelevu	2021	New Zealand, USA	Giving insight into Pacific scholars’ engagement with social media to enact digital vā	Qualitative	Cohort study, talanoa	Convenience sampling (peer network)	Experiences, motivations and methods of Pacific scholar social media use	Thematic analysis, PECAN model	Using PECAN model to focus on tension rather than homogenising findings. Finds that the internet eliminates distance and creates new opportunities for maintaining vā. Pacific online spaces can have counterpublic qualities, facilitating community through shared lived experiences including of marginality. Pacific online spaces create opportunities through vā to reconnect with Pacific ways of knowing and sharing knowledge
Re‐imagining the Dialogic Spaces of Talanoa through Samoan Onto‐ epistemology	J. Matapo, D. Enari	2021	New Zealand, Australia	Discussing talanoa as a tool for Pacific researchers and internet users	Qualitative					At the heart of digital vā is materiality: technology supports the intersubjectivity of Pacific people and extends the possibility for intergenerational and transnational talanoa
Talanoa Moe Vā: Pacific Knowledge‐ sharing and Changing Sociocultural Spaces during COVID‐19	R. Faleolo	2021	Australia	Exploring talanoa and vā within research relationships and how these concepts have changed as they moved online						Talanoa and vā are interconnected research concepts through their mutual emphasis on reciprocity, and each help to build clear and stable online research relationships
The Virtual Faikava: Maintaining Vā and Creating Online Learning Spaces During COVID‐19	T. M. Henry, S. Apo Aporosa	2021	New Zealand	Exploring virtual faikava as a means of mitigating the negative effects of COVID‐19 among Pacific communities	Qualitative	Case study				Virtual faikava strengthened relationships, vā, and well‐being between and of community members during COVID‐19, as real‐time faikava does. Virtual faikava can reveal the importance of interrelated concepts for extending vā online using kava, such as tā and talanoa
Vā at the Time of COVID‐19: When an Aspect of Research Unexpectedly turns into Lived Experience and Practice	A. L. Refiti A. Engels‐ Schwarzpaul, L. Lopesi, B. Lythberg, L. Waerea, V. Smith	2021	New Zealand	Exploring how virtual events can uphold tikanga and vā, and the need for an alternative vā in expected times						COVID‐19 provided a forced backdrop to test digital vā relations. Though there remains much to be navigated in digital space as related to vā, the relation of vā to place, people, and things can help to develop a sense of belonging and grounding during research processes
Navigating the Digital Va‐vā: Centring Moana/Pacific Values in Online Tertiary Settings during COVID‐19	A. M. Fa’aea, S. M. Fonua, C. Chu‐Fuluifaga, J. Ikiua‐Pasi	2021	New Zealand	Exploring how COVID‐19 prompted the authors to re‐focus and re‐think teaching and learning towards Pacific values, mitigating some negative effects of the pandemic	Qualitative	Case study, talanoa				Experiences of online tertiary settings can remind us that values are critical in guiding Pacific ways of being, and should prompt exploration of their enactment in digital and Western educational spaces
A Different Kind of Vā: Spiraling through Time and Space	A. L. Refiti, A. Engels‐ Schwarzpaul, B. Lythberg, V. Smith, L. Waerea	2022	New Zealand	Considering the challenge of navigating digital vā in online and meeting spaces	Qualitative	Case study, talanoa				People have different interpretations and understandings of vā and enact it accordingly. Thus, established relationships are pivotal to how successful online vā can be, and we should focus on identifying the core values of vā that must be sustained when online
Indigenous Education within Urban Contexts and Negotiations in the Diaspora: Talanoa Vā in the Moana	D. T. M. Fa’avae, B. Lealaiauloto, T. Baice, F. Fonua, S.M. Fonua	2022	New Zealand	Exploring education for Pacific people in urban, diasporic contexts	Qualitative	Cohort study, talanoa	Convenience sampling (peer network)	How Indigenous forms of education, knowledge, and tradition are sustained across the diaspora	Thematic analysis of talanoa materials (e.g. emails, Zoom transcripts)	Vā is shaped by participants and their relationship to their wider family, including as informed by cultural values. These cultural and spiritual connections and values are both retained and reconceptualised to maintain links between the diaspora and the homeland through talanoa
Digital Vā: Pacific Perspectives on the Shift from ‘Ordinary Practices’ to ‘Extraordinary Spaces’ During the COVID‐19 Pandemic in Aotearoa/New Zealand	E. S. Fehoko, D. T. M. Fa’avae, A. Tecun, S. A. Siu’ulua	2022	New Zealand	Examining how the quick move to the online world during COVID‐19 impacted three Pacific men and their communities	Qualitative Study	Cohort study, talanoa	Convenience sampling (peer network)	How digital iterations of real‐time cultural practices such as faikava, church‐going, and education are experienced by and impact Pacific people	Thematic analysis of talanoa materials (e.g. emails, Zoom transcripts)	Different Pacific people respond differently to digital vā and depend largely on talanoa to sustain vā online. While elders and large community groups like churches initially struggled with the transition, young and diaspora people were strong navigators of digital vā. Resultantly, vā appeared in unexpected places, such as between families sharing lockdown together, reminding us that digital vā can be useful while exposing some lingering inequities
Engaging and Developing Community in Digital Spaces: Approaches from the Editorial Development Group	O. Karamercan, J. Matapo, O. Kemenarac, D. T. M. Fa’avae, S. Arndt, R. Irwin, E. Kruger, C. Mika, M. Y. A. Bassidou, M. Tesar, P. Del Monte	2022	France, New Zealand, Australia, South Africa, Niger	Offering insight into how digital spaces affect a range of communities, including Pacific people	Qualitative					Digital vā can allow us to re‐think humanist theory and move beyond humanism
e‐Talanoa as an Online Research Method: Extending Vā‐relations across Spaces	D. T. M. Fa’avae, R. Faleolo, E. H. Havea, D. Enari, T. Wright, A. Chand	2022	New Zealand, Australia	Outlining e‐talanoa as a research method and exploring how vā‐relations intertwine with this practice	Qualitative	Talanoa	Convenience sampling (peer network)	Measuring use and meaning of e‐talanoa according to eight questions	Thematic analysis of talanoa data (e.g. Google docs)	Vā is crucial to talanoa, and this remains the case for e‐talanoa. Although e‐talanoa uses anglicised language, focusing on values like clear communication and tauhi vā at the heart of talanoa are critical in maintaining it as a distinctly Pacific online method. However, inequity in digital access is a barrier to e‐talanoa
Rethinking Oceanic‐ Pacific Methods of Data Collection during COVID‐19: Insights From the Field	B. Ofe‐Grant	2022	New Zealand	Offering insight into the challenges researchers faced when collecting data with Pacific people during COVID‐19 and ideas for mitigating these	Qualitative					A multi‐faceted approach to data collection is important in new spaces, especially during crises like COVID‐19 which reinvigorated Pacific people's historical mistrust of researchers. Practices from real‐time data collection can help to establish vā in these cases, such as meeting with participants prior to talanoa and using prayer
Navigating Digital Inclusion and the Digital Vā among Niue Mamatua through the Provision of Mobile Phones During COVID‐19	A. Matenga‐ Ikihele, F. Fa’alu, R. Dobson, J. Fa’alili‐Fidow, M. Roberts, S. Taufa, R. Tuesday, R. Whittaker, J. McCool	2023	New Zealand	Exploring Niuean elders’ ability and willingness to use mobile phones during lockdown, and how they enacted digital vā using this new technology	Qualitative study	Cohort study, tutala	Convenience Sampling (*n* = 12 mamatua)	How mobile phones helped contribute towards digital inclusion and well‐being	Thematic analysis of transcribed audio recordings organised and coded by NVivo 12 software	The provision of mobile phones to elders during COVID‐19 had positive impacts and revealed underlying inequities. Quick access, convenience, and connection to family, church, Niue, the world and health services were beneficial. Cost and visual/physical difficulties presented challenges. To support success, elders recognised the intergenerational support they received, learnt new digital skills, and integrated online space into regular vā practices with family

## 3 Results

Of the 17 included studies, 10 were empirical studies and seven were framework studies, all focused on conceptualising and exploring how real‐time vā practices translate and operate online.

### 3.1 Conceptions of Vā and Digital Vā

Vā was consistently described as the space between things, people and points in time (Fa’aea et al. 2021; Fehoko et al. 2022; Henry and Aporosa 2021; Koya Vaka’uta 2017; Laulaupea’alu 2021; Matapo and Enari 2021; Refiti et al. 2021; Tielu 2016). It is a relational ontology facilitating relationships between all beings and things, with a temporal element marking these relationships through ‘spaces‐in‐between’ (Fehoko et al. 2022, p. 310) and being ‘always present in, through, and around us’ (Matapo 2022, p. 3). Understandings of vā are grounded in Indigenous Pacific epistemic frameworks, emphasising interdependence and co‐existence (Fa'avae, Faleolo et al. 2022; Fa'avae, Lealaiauloto et al. 2022; Faleolo 2021; Fehoko et al. 2022; Koya Vaka’uta 2017; Matapo 2022; Matapo and Enari 2021; Refiti et al. 2021).

The importance of actively maintaining vā was reiterated, often articulated as a practice nurtured through following pre‐established rules of social engagement (Fa’aea et al. 2021; Fa'avae, Faleolo, et al. 2022; Fa'avae, Lealaiauloto, et al. 2022; Faleolo 2021; Fehoko et al. 2022; Henry and Aporosa 2021; Koya Vaka’uta 2017; Laulaupea’alu 2021; Matenga‐Ikihele et al. 2023; Ofe‐Grant 2022; Thomsen et al. 2021; Tielu 2016). This active component of vā was often described as formulated around and guided by Tongan tauhi vā (Fa'avae, Lealaiauloto et al. 2022; Faleolo 2021; Fehoko et al. 2022; Koya Vaka’uta 2017; Laulaupea’alu 2021) and Samoan teu le va (Fa'avae, Lealaiauloto, et al. 2022; Faleolo 2021; Koya Vaka’uta 2017; Matenga‐Ikihele et al. 2023; Thomsen et al. 2021), both meaning to nurture social relations. However, several studies emphasised that, like vā itself, these concepts tend to have pan‐Pacific variants and are based around shared values of building and sustaining positive relationships between oneself, one's relatives and one's community. As the space between, vā relates rather than separates and the conceptual language surrounding vā functions similarly (Fa’aea et al. 2021; Fa'avae, Lealaiauloto, et al. 2022; Fehoko et al. 2022; Koya Vaka’uta 2017).

Though a distinct conception of vā was outlined, *digital* vā was less clear. Contestation appeared regarding whether digital vā simply constituted an online version of vā or whether it was a distinct concept and/or practice. In eight studies, digital vā was understood as the adaptative moving of real‐time vā into online space, responsive to spatio‐temporal changes related to migration and new communication technologies (Enari and Matapo 2020; Fa’aea et al. 2021; Fa'avae, Lealaiauloto, et al. 2022; Fehoko et al. 2022; Matapo and Enari 2021; Matenga‐Ikihele et al. 2023; Refiti et al. 2021; Thomsen et al. 2021). In understanding vā as the environment wherein interaction happens, these studies applied this materiality to an online context, rendering technology part of the vā assemblage (Matapo 2022; Matapo and Enari 2021; Thomsen et al. 2021). The relational nature of vā was recognised as affording it a natural changeability. For instance, COVID‐19 catalysed a new wave of digital vā research (Enari and Matapo 2020; Faleolo 2021; Fehoko et al. 2022; Henry and Aporosa 2021), but many Pacific people had engaged digital space to maintain vā well before being forced online by the pandemic (Enari and Matapo 2020; Fehoko et al. 2022). In turn, digital vā was conceived of as an opportunity for the Indigenisation of online space (Enari and Matapo 2020; Refiti et al. 2021) and an anti‐colonial tool of reconnection and voice (Fa'avae, Lealaiauloto, et al. 2022; Matapo and Enari 2021; Thomsen et al. 2021) following the evolving social needs and desires of Pacific people.

Contrastingly, six studies were identified that understood digital vā as ‘distinct from offline space, place and time’ (Koya Vaka’uta 2017, p. 6). While acknowledging that digitising vā was valuable for connecting the diaspora and giving voice to marginalised people, these studies articulated the experience of digital vā as different from the experience of real‐time vā (Fa'avae, Faleolo, et al. 2022; Fa'avae, Lealaiauloto, et al. 2022; Fehoko et al. 2022; Matenga‐Ikihele et al. 2023; Refiti et al. 2021; Thomsen et al. 2021). Though practices for nurturing real‐time vā were able to be mimicked online, establishing the same opportunities for these interactions was felt to be difficult (Fehoko et al. 2022; Koya Vaka’uta 2017; Henry and Aporosa 2021). For instance, Matenga‐Ikihele et al. (2023) noted that real‐time vā practices are traditionally passed down by elders, but modern digital vā practices are instead taught to elders by youth, and Koya Vaka’uta (2017) characterised digital spaces as increasingly ordered by individualism and consumerism. Thus, the different materiality of digital vā was understood as challenging usual conceptions and enactment of vā, requiring the development of new practices for a new space (Fa’aea, et al. 2021; Faleolo 2021; Fehoko et al. 2022; Henry and Aporosa 2021; Matenga‐Ikihele et al. 2023; Refiti et al. 2021)—a ‘modern form of wayfinding’ (Henry and Aporosa 2021, pp. 190–191).

### 3.2 Frameworks Used in Studies of Digital Vā

A variety of conceptual frameworks utilised in relation to digital vā were identified. Nine studies utilised talanoa, a relational methodology that focuses on informal conversations with research participants and centralises co‐constructive knowledge‐making. Though each Pacific cultural group conducts talanoa differently, talanoa always requires that vā be created and maintained between participants to be effective and, in turn, the reciprocity of talanoa nurtures vā (Fa'avae, Faleolo, et al. 2022; Faleolo 2021; Fehoko et al. 2022; Henry and Aporosa 2021; Laulaupea’alu 2021; Ofe‐Grant 2022; Refiti et al. 2021; Thomsen et al. 2021). Talanoa was also identified as a means to tauhi vā (Fehoko et al. 2022) and teu le va (Ofe‐Grant 2022). In recognising the interdependence of talanoa and vā, Fa’avae, Faleolo, et al. (2022) and Fehoko et al. (2022) conceive of talanoa‐vā as a decolonial framework enabling the critical interrogation of relationship‐ and knowledge‐building through engaging points of tension and complexity, something particularly useful in online contexts.

Less often, other Indigenous theoretical frameworks were identified as relevant to digital vā. Fa’avae, Faleolo, et al. (2022) discussed Samoan Indigenous Reference as a framework for exploring and sustaining the ethical and genealogical relations at the core of vā when moving across different times and materialities. The study supplements this approach with reference to the Vā Moana digital platform for exploring educational practices across diaspora. With more attention to digital vā specifically, Fa’aea et al. (2021) described the use of *‘*Ulungaanga faka‐Tonga Fonu, a teaching model centred on Tongan behavioural concepts, to build vā through values‐sharing in an online Western educational space. Similar to Fa’aea et al.'s emphasis on promoting Pacific values to enable better navigation of digital vā, Koya Vaka’uta (2017) explores values theory as a framework for articulating how digital spaces may be organised by values that contrast Indigenous values, necessitating that digital vā be navigated with care.

Three established frameworks related to well‐being were also identified. Matenga‐Ikihele et al.'s (2023) study of Niuean elders during COVID‐19 noted that the restorative effect of digital vā could be read alongside the Fonofale Model. Further, the scholars also drew on two recently developed national frameworks, Better Later Life: He Orana Kaumātua 2019–2024 and Ola Manuia: Pacific Health and Well‐being Action Plan 2020–2025, to explain the connective role of cellphones for elders, suggesting that considering digital inclusion and exclusion is essential when exploring digital vā.

### 3.3 Concepts Used Alongside Digital Vā

A range of supplementary concepts were identified as relevant to digital vā during the review. Pacific values and practices appeared often as ways of exploring how the conditions for vā may or may not be possible to create online (Fa’aea et al. 2021; Fa'avae, Faleolo, et al. 2022; Henry and Aporosa 2021; Koya Vaka’uta 2017; Matenga‐Ikihele et al. 2023; Ofe‐Grant 2022; Thomsen et al. 2021). A range of ethnicity‐specific relational concepts with pan‐Pacific relevance were noted as essential for maintaining vā while online, most frequently being talanoa, alongside the Samoan, Tongan, Fijian and Niuean variants of service, love and respect (Fa’aea et al. 2021, Fa'avae, Faleolo, et al. 2022; Faleolo 2021; Fehoko et al. 2022; Henry and Aporosa 2021; Koya Vaka’uta 2017; Laulaupea’alu 2021; Matapo and Enari 2021; Matenga‐Ikihele et al. 2023; Ofe‐Grant 2022; Refiti et al. 2021). Similarly, the importance of engaging values‐based practices in online spaces to create and nurture vā was reiterated, with suggestions that faikava (regular kava gathering) (Fehoko et al. 2022; Henry and Aporosa 2021; Laulaupea’alu 2021) and prayer (Refiti et al. 2022) were aspects of real‐time vā that translated well to digital contexts. There was broad consensus as to the criticality of retaining the value foundations of vā in online spaces, especially for younger generations (Fa'avae, Faleolo et al. 2022; Faleolo 2021; Koya Vaka’uta 2017; Refiti et al. 2022).

Less frequently, understanding of digital vā was developed by emphasising Pacific epistemology. Digital vā is an alternative to and critical of Western humanist knowledge systems that ‘evokes us to think through digital entanglements of human and non‐human worlds’ (Matapo 2022, p. 3). In this way, virtual spaces hold potential for the decolonial or postcolonial development of Pacific ontology, something noted as particularly applicable to education contexts as a frontier of digital vā (Enari and Matapo 2020; Fa’aea et al. 2021; Fa'avae, Faleolo et al. 2022; Fehoko et al. 2022). In this vein, eight studies were establishing digital vā by building on and adapting concepts important to real‐time vā. This process happened in particular around notions of time and place. For instance, Henry and Aporosa 2021 and Fa’aea et al. (2021) raised the Tongan notion of tā (beats and rhythms between people and things) as something still being formed in the digital realm. Fehoko et al. (2022) and Fa’avae, Lealaiauloto, et al. (2022) expanded on tā‐vā by suggesting it laid ground for developing relational potential across generations. Focus on place was sometimes oriented around notions of Pacific mobility (Fa'avae, Faleolo, et al. 2022; Fa'avae, Lealaiauloto et al. 2022), with Thomsen et al. (2021) raising the idea of Moana publics to consider what online places can offer to Pacific people. In other cases, focus on place was channelled through vahaope, a Tongan term for the internet meaning beyond the boundaries of physical locale (Henry and Aporosa 2021), a place with e‐culture and e‐practices distinct from real time (Koya Vaka’uta 2017). E‐talanoa was discussed in a similar way, providing opportunities for reshaping traditional knowledge in response to contemporary Pacific modes of connection (Fa'avae, Faleolo, et al. 2022; Faleolo 2021).

Social scientific ideas were also applied to digital vā (Fa'avae, Faleolo et al. 2022; Fa'avae, Lealaiauloto et al. 2022). In particular, the digital divide (Ofe‐Grant 2022; Refiti et al. 2022) and digital inclusion and exclusion (Fa'avae, Faleolo et al. 2022; Fehoko et al. 2022; Matenga‐Ikihele et al. 2023) were utilised to consider possible constraints for engaging digital vā. Despite a general optimism towards the ability to enact vā principles in online spaces, inequitable access to digital technology was widely noted as a major barrier to the development of digital vā (Fa'avae, Faleolo, et al. 2022; Fehoko et al. 2022; Koya Vaka’uta 2017; Matenga‐Ikihele et al. 2023).

### 3.4 Well‐Being and Digital Vā

Generally, online spaces were considered to have positive and negative effects on well‐being. At the broadest level, studies considered positive social connection as central to Pacific well‐being (Fa’aea et al. 2021; Fa'avae, Faleolo, et al. 2022; Fa'avae, Lealaiauloto, et al. 2022; Faleolo 2021; Fehoko et al. 2022; Henry and Aporosa 2021; Koya Vaka’uta 2017; Laulaupea’alu 2021; Matapo and Enari 2021; Matenga‐Ikihele et al. 2023; Ofe‐Grant 2022; Thomsen et al. 2021; Tielu 2016; Refiti et al. 2022). Both Koya Vaka’uta (2017) and Matapo and Enari (2021) raised concerns about the individualism present in and encouraged by online spaces, leading to negative effects on well‐being such as gambling, obesity, addiction and mental illness (Fehoko et al. 2022) or a disconnected sense of cultural and personal identity (Fehoko et al. 2022; Koya Vaka’uta 2017). Further, (Laulaupea’alu 2021) addressed the increased opportunity online for behaviour that would interrupt vā, such as online scams or cheating during online exams.

Several studies identified how specific practices enacted online contributed to the development of digital vā with positive effects on well‐being. On a small scale, Refiti et al. (2022) found that e‐inoi (prayer) was able to satisfactorily replace song as a way of increasing social and spiritual well‐being during COVID‐19. Three studies noted that online faikava resulted in health benefits, including mental clarity, relaxation and social connection (Fehoko et al. 2022; Henry and Aporosa 2021; Laulaupea’alu 2021). Finally, Enari and Matapo (2020) suggested that online pedagogy had a collective benefit for Pacific families beyond only the learner.

On a structural scale, Thomsen et al. (2021) argued that Pacific online community‐building generated empowering digital vā through collectivity and shared resistance to marginalisation in popular digital spaces, sometimes translating into real‐time vā. Matenga‐Ikihele et al. (2023) noted that digital inclusion helped alleviate pandemic‐induced loneliness and anxiety among Niuean elders, built real‐time intergenerational bonds between families and facilitated better access to healthcare services. Despite occasional unfamiliarity, shared experimentation with cultural practices in digital space that centred around vā ethics created new potential for enhancing social connection (Fa'avae, Lealaiauloto et al. 2022; Refiti et al. 2022).

## 4 Discussion

Though we initially aimed to review the concept of digital vā as related to online gaming and Pacific youth well‐being, no existing research that tied these concepts together was identified by our scoping review. Instead, we have examined digital vā across a wider range of online contexts. With focus on digital vā largely responsive to the context of COVID‐19, the body of literature we reviewed reflected a disregard for pre‐pandemic social use of online platforms by diaspora Pacific. Further, seemingly owing to a sense that youth are more familiar with digital technologies, the studies reviewed generally failed to account for the unique experiences of youth in digital spaces and how they navigate them differently than Pacific adults and elders.

Across the reviewed studies, there was a unanimous understanding of vā as an intangible relational ontology that forms the space‐time in and around which social interactions occur. Vā is a process requiring maintenance, including through enacting and embodying a range of Pacific cultural practices and values (Fa’aea et al. 2021; Fa'avae, Faleolo, et al. 2022; Fa'avae, Lealaiauloto, et al. 2022; Faleolo 2021; Fehoko, et al. 2022; Henry and Aporosa 2021; Koya Vaka’uta 2017; Laulaupea’alu 2021; Matenga‐Ikihele et al. 2023; Ofe‐Grant 2022; Thomsen et al. 2021; Tielu 2016). However, *digital* vā was more contentious, and the review identified a range of ideas about what digital vā is, means and how it should be used. For some, digital vā has been an organic development per the changing needs of Pacific people and presents opportunities for connection (Enari and Matapo 2020; Fa’aea et al. 2021; Fa'avae,  Lealaiauloto, et al. 2022; Fehoko et al. 2022; Matapo and Enari 2021; Matenga‐Ikihele et al. 2023; Refiti et al. 2021; Thomsen et al. 2021). For others, digital vā has been experienced differently to real‐time vā, requiring consistent attention to established vā ethics (Fa'avae, Faleolo, et al. 2022; Fa'avae, Lealaiauloto, et al. 2022; Fehoko et al. 2022; Henry and Aporosa 2021; Matenga‐Ikihele et al. 2023; Refiti et al. 2021; Thomsen et al. 2021). Attempts to navigate this tension underscore the necessity of youth‐focused research into how Pacific youth engage their cultures in a changing world (HQSC 2021; Veukiso‐Ulugia et al. 2024).

In particular, the response to COVID‐19 raises further questions about conflicted approaches to digital vā. Despite indications that the concept of vā had already been evolving before the pandemic (Enari and Matapo 2020; Koya Vaka’uta 2017; Tielu 2016), most of the literature reviewed was written during or after 2020. Though scholars firmly rejected the notion that digital vā emerged by force of the pandemic, some of the trepidation towards digital vā by those writing after its onset might result from having been denied the ability to develop digital vā naturally and flexibly. Here, we suggest that having not experienced the pandemic‐induced transition to frequent technology use so harshly, youth may perceive a less distinct divide between vā and digital vā—or even different conceptions of digital vā entirely.

Talanoa was the most common concept identified and conceptualised alongside vā. Being a co‐constructive and relational methodology and practice, talanoa has a mutually constitutive relationship with vā—one cannot exist without the other (Fa'avae, Faleolo, et al. 2022; Faleolo 2021; Fehoko et al. 2022; Henry and Aporosa 2021; Laulaupea’alu 2021; Ofe‐Grant 2022; Refiti et al. 2021; Thomsen et al. 2021). However, as Matenga‐Ikihele et al. (2023) point out in their study with Niuean elders, talanoa is unsuitable for all Pacific communities. As different ways of building vā must be nurtured and developed in real‐time according to community needs, the same must happen in online spaces. Broad notions of e‐talanoa (Fa'avae, Lealaiauloto, et al. 2022; Faleolo 2021; Koya Vaka’uta 2017) and smaller scale practices like e‐inoi (Refiti et al. 2022) represent new ways in which digital vā is supported and grown through responsive online practices. As Thomsen et al. (2021) note, Pacific people use online social spaces creatively, and this is what draws out the potential of the technology. With different and longer‐standing relationships to digital spaces and technology (HQSC 2021; Koya Vaka’uta 2017; Lopesi (2018), youth may have unique understandings of how to engage or build digital vā.

Though most scholars implied that positive vā relations are essential to Pacific well‐being, others conceptualised this more concretely. By considering how digital vā supported the well‐being of Niuean elders throughout COVID‐19, Matenga‐Ikihele et al. (2023) argued that the positive impacts felt through maintaining vā connections bring to fruition the values of Fonofale. However, exploring Fonofale and other health frameworks throughout the review fails to link the benefits or disadvantages of digital vā to Pacific youth, despite indication of its relevance: Enari and Matapo (2020) suggest that online education has developed digital vā relations beyond the class to family and community; Laulaupea’alu (2021) explores the negative effect on mental well‐being for Pacific university students as a result of pandemic isolation requirements; and Matenga‐Ikihele et al. (2023) find that unlike real‐time vā practices, digital vā practices are taught to elders by youth. For example, considering Matenga‐Ikihele et al.'s finding that the intergenerational experience of online spaces strengthened familial vā relations offline, we ask whether the fluid space‐time arrangement of vā means that digital vā offers youth the unique opportunity to enhance collective well‐being without disturbing the traditions of real‐time vā. Existing literature lacks insight into how digital spaces shape the ability of Pacific youth to connect with their families, cultures and communities in new ways, and how this in turn shapes their well‐being.

In particular, the specific digital platforms explored by existing literature are limited. While Thomsen et al. (2021) focuses on Twitter (now X) and others consider the implications of various communication technologies for teachers/students or researchers/participants (Enari and Matapo 2020; Fa’aea et al. 2021; Fa'avae, Faleolo, et al. 2022; Fa'avae, Lealaiauloto, et al. 2022; Laulaupea’alu 2021; Matapo and Enari 2021; Ofe‐Grant 2022), little attention is paid to newer spaces which are more familiar to young people. For instance, it would be of interest to explore new online social spaces, such as player versus player gaming that incorporates e‐talanoa over microphones and shared online spaces. Gaining a fuller understanding of how digital vā is developed and maintained in new digital spaces would further support insight into how digital vā affects Pacific youth. This review therefore supports recent calls to consider Pacific youth understandings of digital spaces from their own perspectives (HQSC 2021; Veukiso‐Ulugia et al. 2024).

After the completion of this scoping review, additional research into digital vā has been published. Meiklejohn–Whiu (2024) employed the notion of vā and digital vā as a means of exploring the potential of digital and counter storytelling in supporting Moana educational identity development. More recently, Meiklejohn–Whiu and Jesson (2026) explored how digital learning design could create relational spaces for students, finding that culturally responsive digital environments underpinned by relational principles support meaningful engagement and identity development for Moana students. While these studies are outside of the scoping review, we acknowledge this work and the important contribution it makes to emerging discussions about digital vā.

### 4.1 Indigenous Pacific Reflections

The use of scoping reviews as a methodological tool to review literature in an emerging field is useful for providing insight into the published field and identifying academic research gaps. We use the term ‘published field’ purposefully here: An idea not being published does not mean it is not being discussed, thought about, theorised or considered outside of academic spaces. While helpful, scoping reviews also perpetuate colonial hierarchies of knowledge, reinforcing the privileging of published research and indexed journals often over community‐based scholarship and student research. Indigenous Pacific scholars and wider communities understand that community is a key site of Indigenous theorisation (Smith 2012; Wilson 2008). Therefore, while this scoping review has provided a starting point for academic discussion, it does not entirely reflect what digital vā means or how it is understood, conceptualised or employed.

## 5 Conclusion

This scoping review has established that real‐time vā and digital vā are intrinsically linked. While there is a resounding indication that mindful navigation should be exercised in this new vā environment, digital vā is here to stay, especially in the wake of COVID‐19. Indisputably, ‘virtual sociocultural spaces have become increasingly important to Pacific knowledge‐sharing’ (Fa’aea et al. 2021, p. 126) and should be taken seriously as an object of inquiry. In acknowledging that Pacific youth experience a particular understanding of and relationship to digital space, examining their specific uses of new spaces like online games can measure notions of vā and talanoa to understand how youth interact with Pacific cultural practices as they are transferred and enacted online. As with any area pertinent to community well‐being, youth experience and voice are critical in gaining a more transparent and balanced understanding of what digital vā means and how it is nurtured.

## Funding

This work was funded by the University of Auckland, Faculty of Education and Social Work, Research Development Fund #3726364.

## Conflicts of Interest

The authors declare no conflicts of interest.

## Data Availability

Data sharing not applicable to this article as no datasets were generated or analysed during the current study.
